# IDENTIFY THE BARRIERS THAT FAMILY CAREGIVERS FACE IN MANAGING PATIENTS’ PAIN: A LITERATURE REVIEW

**DOI:** 10.1093/geroni/igz038.2631

**Published:** 2019-11-08

**Authors:** Nai-Ching Chi, Ying-Kai Fu, Emelia Barani, Elizabeth Summerson

**Affiliations:** 1 University of Iowa, Iowa city, Iowa, United States; 2 University of Washington, Seattle, Washington, United States

## Abstract

Most Americans enrolled in palliative and hospice care receive care at home and thus rely on their family caregivers (FCs) to manage their pain. However, FCs encounter many barriers to pain management. Previous studies focused on barriers faced by FCs of cancer patients. However, as palliative and hospice care evolves, the populations and diagnoses become diverse, and more barriers have been identified. Hence, the purpose of this study is to comprehensively investigate FCs’ barriers to pain management. A literature review was conducted to search available articles published before June 2018 in databases including PubMed, CINAHL, PsycINFO, and Scopus. Search strategies used index and keyword methods. The inclusion criteria were peer-reviewed, research studies published in English that explored barriers that FCs faced in managing pain. Eighty-six studied were identified: 76% of the studies focused on cancer pain and 6% focused on dementia pain. The identified barriers included: (1) caregivers’ limited knowledge in drug/non-drug pain management and verbal/non-verbal pain assessment; (2) caregivers’ issues (e.g. function, fear of analgesic, misbeliefs in pain management); (c) caregivers’ organizational skills (treatment recording and tracking); (d) patients’ issues (e.g. inability to verbalize pain); (e) communication issues with care teams. This is one of the few literature reviews that comprehensively investigate the barriers that family caregivers experience beyond cancer pain management. The results can be used to develop a screening questionnaire for palliative and hospice providers to assess and resolve FCs’ barriers to pain management, thereby improving the quality of pain management and patients’ and FCs’ outcomes.

